# Evaluation of novel AI‐based extended field‐of‐view CT reconstructions

**DOI:** 10.1002/mp.14937

**Published:** 2021-05-31

**Authors:** Gabriel Paiva Fonseca, Matthias Baer‐Beck, Eric Fournie, Christian Hofmann, Ilaria Rinaldi, Michel C Ollers, Wouter J.C. van Elmpt, Frank Verhaegen

**Affiliations:** ^1^ Department of Radiation Oncology (MAASTRO) GROW School for Oncology and Developmental Biology Maastricht University Medical Centre+ Maastricht 6229 ET The Netherlands; ^2^ Siemens Healthcare GmbH Forchheim Germany

**Keywords:** CT imaging, extended field of view, HDFoV, radiotherapy, 3D printing

## Abstract

**Purpose:**

Modern computed tomography (CT) scanners have an extended field‐of‐view (eFoV) for reconstructing images up to the bore size, which is relevant for patients with higher BMI or non‐isocentric positioning due to fixation devices. However, the accuracy of the image reconstruction in eFoV is not well known since truncated data are used. This study introduces a new deep learning‐based algorithm for extended field‐of‐view reconstruction and evaluates the accuracy of the eFoV reconstruction focusing on aspects relevant for radiotherapy.

**Methods:**

A life‐size three‐dimensional (3D) printed thorax phantom, based on a patient CT for which eFoV was necessary, was manufactured and used as reference. The phantom has holes allowing the placement of tissue mimicking inserts used to evaluate the Hounsfield unit (HU) accuracy. CT images of the phantom were acquired using different configurations aiming to evaluate geometric and HU accuracy in the eFoV. Image reconstruction was performed using a state‐of‐the‐art reconstruction algorithm (HDFoV), commercially available, and the novel deep learning‐based approach (HDeepFoV). Five patient cases were selected to evaluate the performance of both algorithms on patient data. There is no ground truth for patients so the reconstructions were qualitatively evaluated by five physicians and five medical physicists.

**Results:**

The phantom geometry reconstructed with HDFoV showed boundary deviations from 1.0 to 2.5 cm depending on the volume of the phantom outside the regular scan field of view. HDeepFoV showed a superior performance regardless of the volume of the phantom within eFOV with a maximum boundary deviation below 1.0 cm. The maximum HU (absolute) difference for soft issue inserts is below 79 and 41 HU for HDFoV and HDeepFoV, respectively. HDeepFoV has a maximum deviation of −18 HU for an inhaled lung insert while HDFoV reached a 229 HU difference. The qualitative evaluation of patient cases shows that the novel deep learning approach produces images that look more realistic and have fewer artifacts.

**Conclusion:**

To be able to reconstruct images outside the sFoV of the CT scanner there is no alternative than to use some kind of extrapolated data. In our study, we proposed and investigated a new deep learning‐based algorithm and compared it to a commercial solution for eFoV reconstruction. The deep learning‐based algorithm showed superior performance in quantitative evaluations based on phantom data and in qualitative assessments of patient data.

## Graphical abstract

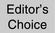

## 1 INTRODUCTION

Radiotherapy treatment plans are based on medical imaging from which CT is the most used technique for both tissue delineation and dose calculation.^1^, ^2^ Modern dose calculation algorithms can precisely calculate dose distributions and optimize treatment plans to deliver a highly conformal dose to the patient using material composition and density extracted from CT images as input.^3^, ^4^, ^5^ In addition to dose calculation, CT images are often used for manual and automated tissue delineation,^6^ surface tracking during treatment,^7^ prediction on portal imaging and treatment verification.^8^ Therefore, the accuracy of CT reconstruction regarding body contours and accurate Hounsfield unit (HU) can affect several aspects of radiotherapy treatments.

Modern CT scanners typically have a scan field‐of‐view (sFoV) of 50 to 70 cm, and in most cases the patient boundary is well within the sFoV. However, in certain cases, for example, in patients with higher body mass index (BMI) or when using a positioning device such as a breast board, part of the patient’s anatomy extends beyond the sFoV. The limited data in the regions outside the sFoV often lead to inaccurate patient geometry and reduced accuracy of the Hounsfield unit (HU) in the extended FoV region (eFoV). To overcome this, eFoV algorithms have been developed^9^, ^10^, ^11^ and constantly been improved over the years to increase the accuracy of reconstruction outside the sFoV.^12^, ^13^, ^14^, ^15^ Most of the methods apply a form of extrapolation to the truncated data in the projection space aiming for a realistic approximation of the geometry outside sFoV.^14^ However, image artifacts and geometrical uncertainties of the order of centimeters^12^ are limiting factors.

Deep learning methods have achieved excellent results in several fields including image processing including the reduction of cupping artifacts,^16^ and denoising of low dose CT.^17^ Most recent advances in eFoV reconstruction are also related to the usage of neural networks to improve the results in case of truncated CT data.^15^, ^16^, ^18^ While the algorithm described by Han et al.^16^ implements a neural network‐based detruncation algorithm for ROI tomography, Fournié et al.^18^ proposed a direct and image‐based method that uses a U‐Net to estimate the CT image also in the regions beyond the sFoV. Huang et al.^19^ proposed a combination of a U‐Net with an iterative reconstruction algorithm based on total variation (TV) minimization for eFoV reconstruction. The HDeepFoV algorithm that is proposed in this paper builds up on the findings of Fournié et al.^18^ With HDeepFoV the network estimate is used to extrapolate the measured projection data into the eFoV region, followed by a direct filtered back projection reconstruction. As compared to the algorithm proposed by Huang et al.^19^ HDeepFoV has a reduced overall complexity due to the absence of an iterative TV framework.

Regardless of the reconstruction method (extrapolation or deep learning), there is limited information about eFoV uncertainties. Cheung et al.^12^ performed a detailed evaluation of three commercial algorithms using a custom phantom as reference. Although some data on eFoV uncertainties were provided, only a single central slice of the phantom was evaluated while our clinical experience indicates eFoV reconstruction can vary significantly depending on the treatment site and even between consecutive slices from a CT sequence. In addition, the software version (from 2013) used by Cheung et al.^12^ for the reconstruction of patient cases no longer corresponds to the current state‐of‐the‐art.

This study describes the development of a new approach that uses a convolutional neural network (HDeepFoV) for eFoV reconstruction. The developed method is described and compared to a state‐of‐the‐art eFoV reconstruction algorithm (HDFoV) that is implemented in current Siemens Healthineers CT scanners. An in‐house three‐dimensional (3D) printed phantom, based on the dimensions of a patient, for which eFoV was necessary, was developed and manufactured for this application. The phantom was used for quantitative comparisons while patient cases were qualitatively evaluated by experts. To the best of our knowledge, this is the first study to describe such a novel eFoV reconstruction approach validated with a realistic phantom and evaluated for a range of clinical patients.

## 2 MATERIALS AND METHODS

This section describes two eFoV algorithms (state‐of‐the‐art and novel approach) and their evaluation in a clinical radiotherapy scenario using quantitative (3D printed phantom) and qualitative (patient cases) metrics.

### 2.1 Algorithms for extended field‐of‐view reconstruction

#### 2.1.1 State‐of‐the‐art algorithm: HDFoV

**Fig. 1:**
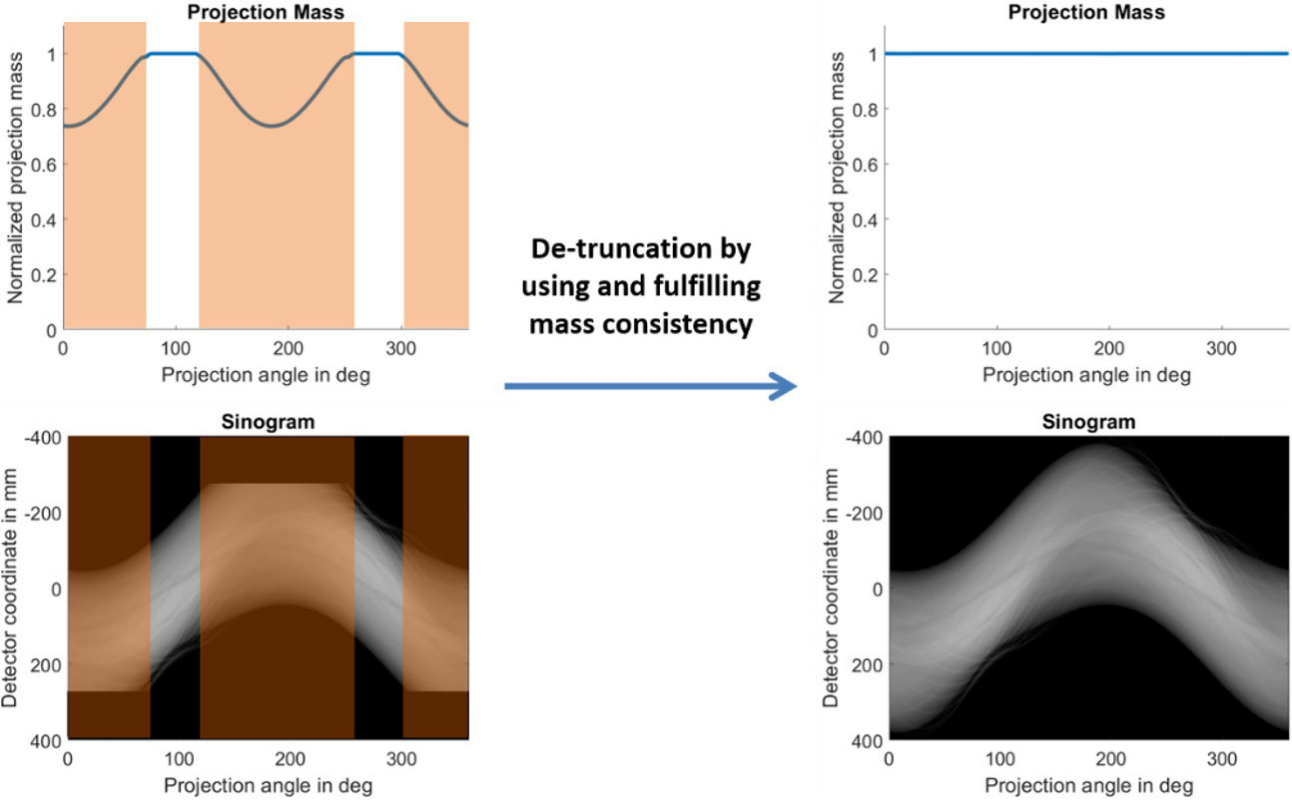
Sinogram detruncation to ensure mass consistency across all the projections. Left: For truncated data the normalized projection mass drops below 1. Right: After extrapolation the mass consistency is fulfilled and the normalized projection mass is constant over all projections.

The HDFoV algorithm for eFoV reconstruction consists of two steps. First, an initial estimate of the image is reconstructed by using the mass consistency condition^20^, ^21^ as described by Hsieh et al.^14^ But instead of using the water cylinder approach for extrapolating the missing data in truncated projections, HDFoV uses a cosine‐shaped function for the extrapolation.^15^ The basic principle of this first step in the HDFoV algorithm is summarized in Fig. 1.

In the second step of HDFoV a first estimate of the image is reconstructed from the extrapolated data. The resulting image is then binarized to get an estimate of the object boundaries. To ensure smooth outer contours a 3D Gaussian low pass filter is applied to the image prior to binarization. The binary object mask is then forward projected using the geometry of the original CT scan and in the last step the measured data are extrapolated by using the forward projections from the binary image. The final image is reconstructed from these data. Note that the measured data within the sFoV itself stay untouched by the extrapolation and the simulated data are only used in regions beyond the extent of the sFoV where no measured data are available. HDFoV (version Som/7 VB20) commercially released in 2019 was used in this study.

#### 2.1.2 Proposed deep learning‐based approach: HDeepFoV

**Fig. 2:**
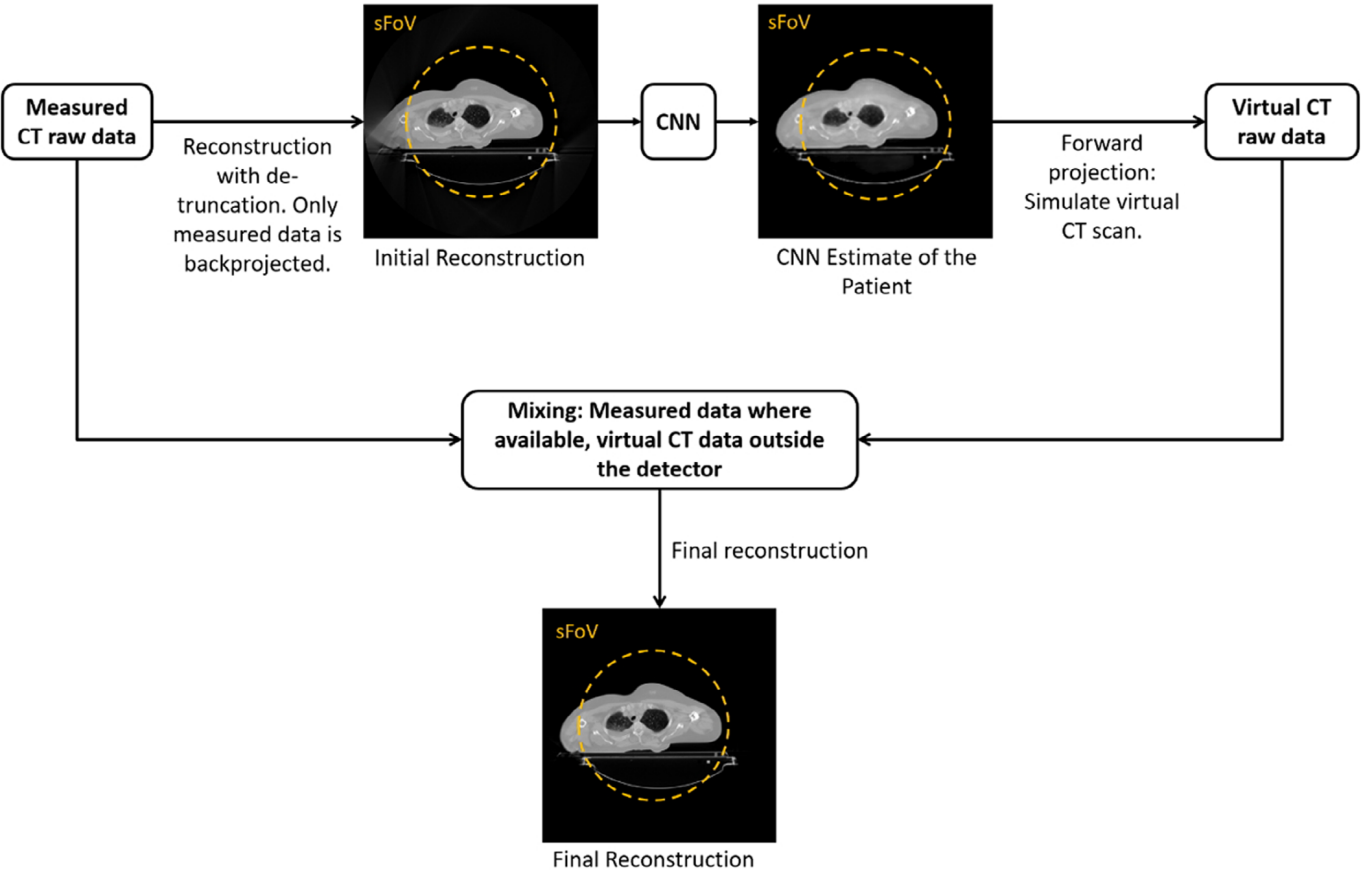
Layout of the HDeepFoV algorithm for extended field‐of‐view reconstruction.

The here proposed HDeepFoV algorithm for eFoV reconstruction is a combination of the deep learning‐based eFoV reconstruction^18^ and the HDFoV algorithm described in the preceding section. In contrast to HDFoV, the proposed HDeepFoV algorithm uses a method similar to the one described by Fournié et al.^18^ to get an initial estimate of the scanned object in the eFoV domain. Furthermore, the binarization step is dropped in HDeepFoV and the forward projection is performed directly on the output images of the neural network. As in the HDFoV algorithm, in the final step of HDeepFoV the data from the forward projection are combined with the measured data inside the sFoV of the CT scanner. A flowchart of the HDeepFoV algorithm is depicted in Fig. 2.

In a previous study^18^ the final images are a combination of the network estimate and a standard reconstruction of the measured data in the sFoV region. The combination is done in image space where the network results are used only in the eFoV region while the sFoV region is taken from the standard reconstruction. To be able to reconstruct an image in the sFoV region without severe truncation artifacts a linear data extrapolation was applied to the measured data. Nevertheless, due to the simple linear detruncation approach, the sFoV reconstructions still contained some remaining truncation artifacts which lead to a potential contrast step at the transition from the eFoV to the sFoV region. To reduce this an empirical scaling function was applied in the image domain. By using the proposed HDeepFoV approach this empirical scaling is not needed as the combination of network data and measured data is done in the projection domain and therefore there is no need to apply some additional detruncation for the reconstruction of artifact‐free sFoV images. Note that the forward projections of the network images do not only provide information about the object in the eFoV region but also serve as truncation artifact correction for the part of the image that is inside the sFoV. Since the data extrapolation based on the network is much more sophisticated than a simple linear extrapolation toward zero, the results inside the sFoV region may profit from this approach in terms of image quality. Nevertheless, the focus of this paper is the performance of the proposed HDeepFoV algorithm in the region beyond the sFoV of the CT scanner and the evaluation of the sFoV region is not within the scope of this paper.

An additional advantage of the proposed HDeepFoV approach, as compared to the method described by Fournié et al.^18^ is that the influence of the network on image sharpness and noise texture in the eFoV region is reduced since the eFoV part of the image does not come directly from the network but the projection data go through the same reconstruction pipeline as the sFoV part of the image (Fig. 2). With HDeepFoV the input images for the CNN can always be reconstructed with a standardized setting for the image quality parameters which are consistent with the image quality parameters used for the training of the CNN. The final HDeepFoV reconstruction can then be done with the desired and customized setting for the image quality parameters. Note that this final reconstruction can be done by any standard CT image reconstruction algorithm, for example, a filtered backprojection type of algorithm and all standard image quality parameters like the reconstruction kernel can be adjusted in this step as usual.

##### 2.1.2.1 Layout and training of the convolutional neural network

**Fig. 3:**
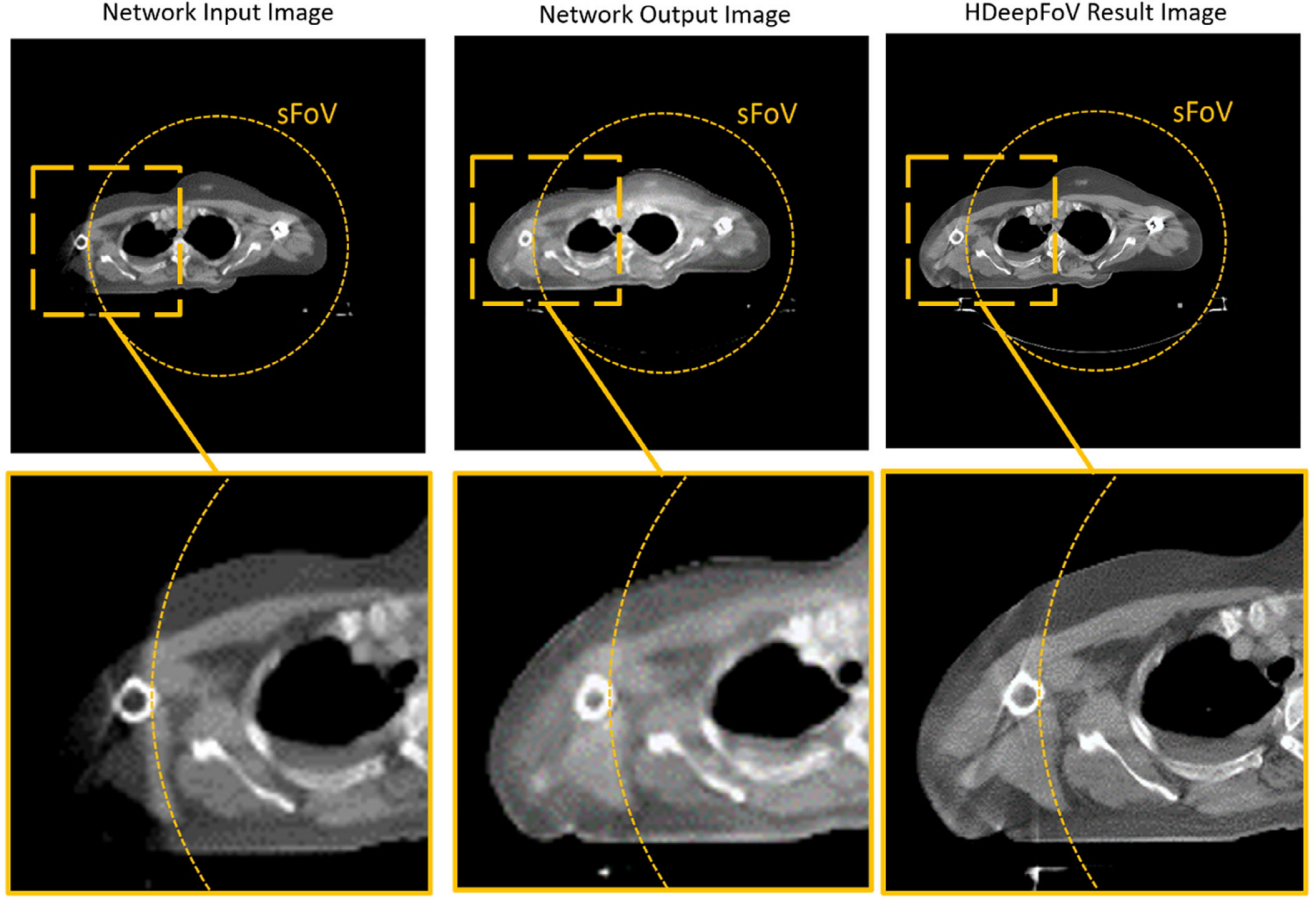
Comparison between network input, network output, and final HDeepFoV result image. In the input and output image of the neural network the noise texture and resolution are altered as compared to the input images due to the reduced matrix size of the images and due to the influence of the neural network. This potential drawback of direct usage of the network results is avoided by the layout of the HDeepFoV algorithm. The final HDeepFoV images show no major visible differences in image resolution and noise texture between the sFoV region and the region outside the sFoV.

For the CNN an U‐Net was chosen. The network used images downsampled to a size of 256 × 256 pixels as input and also the output images of the network were of size 256 × 256. This was done in order to save memory on the GPU so that more training data sets fit into the memory of the GPU. For the training structural dissimilarity^22^ was used as a loss function. Furthermore, the ADAM optimizer^23^ was applied as optimizer and Leaky‐Relu^24^ served as activation function. As can be seen when comparing the sFoV region (measured data for reconstruction) with the region outside the sFoV (data from forward projections of neural network estimate with reduced resolution) in the HDeepFoV images (Fig. 3) the reduced resolution of the network input and output images does not have a major influence on the final results of the HDeepFoV reconstructions which are reconstructed on a 512 × 512 image matrix.

The training of the CNN was done by using simulated CT data sets. In a first step virtual CT raw data were computed by forward projection of clinical CT image data sets. Prior to the forward projection the clinical CT data sets were geometrically altered, that is, they were scaled, rotated, and shifted so that they exceed the sFoV of the CT scanner whose geometry properties were used for the simulation. Here the geometry of the SOMATOM Confidence CT scanner (Siemens Healthineers, Forchheim, Germany) was used. This scanner has a focus to isocenter distance RF of 595 mm and a focus to detector distance, RFD, of 1086 mm which results in a sFoV diameter of 500 mm. The geometrical distortion of the original CT images was limited so that they at least fit into the bore size, i.e. the central opening of the CT scanner. In case of the SOMATOM Confidence this opening has a diameter of 800 mm. The size of the images after the geometrical distortion was set to 800 mm × 800 mm. These images are the ground truth images for the learning process. Based on these ground truth images a forward projection using the geometry of the SOMATOM Confidence CT scanner was done to simulate virtual CT raw data sets with truncation.

The simulated raw data were then reconstructed by a standard filtered backprojection‐based reconstruction algorithm.^25^ Prior to the convolution step of the reconstruction the simulated raw data were extrapolated to avoid truncation artifacts in the reconstructed images. For the extrapolation of the simulated raw data a linear function multiplied by a squared cosine was used. The slope of the extrapolated data was adjusted to the slope of the simulated data at the truncation point, that is, at the first, respectively, last simulated detector pixel. The squared cosine was used to limit the extrapolation range to the dimension of the bore size of the simulated CT scanner. Note that the extrapolated data were only used during the convolution step of the reconstruction. In the final backprojection only data from the initial forward projection were used. The size of the reconstructed field of view was set to 800 mm. These images are the input images for the network used during the training process. Note that by using the method described above an unlimited number of training images can be generated. For the results presented here 60000 pairs of input and ground truth images were used. The training of the network was done using the Keras library.^26^

### 2.2 3D printed phantom

**Fig. 4:**
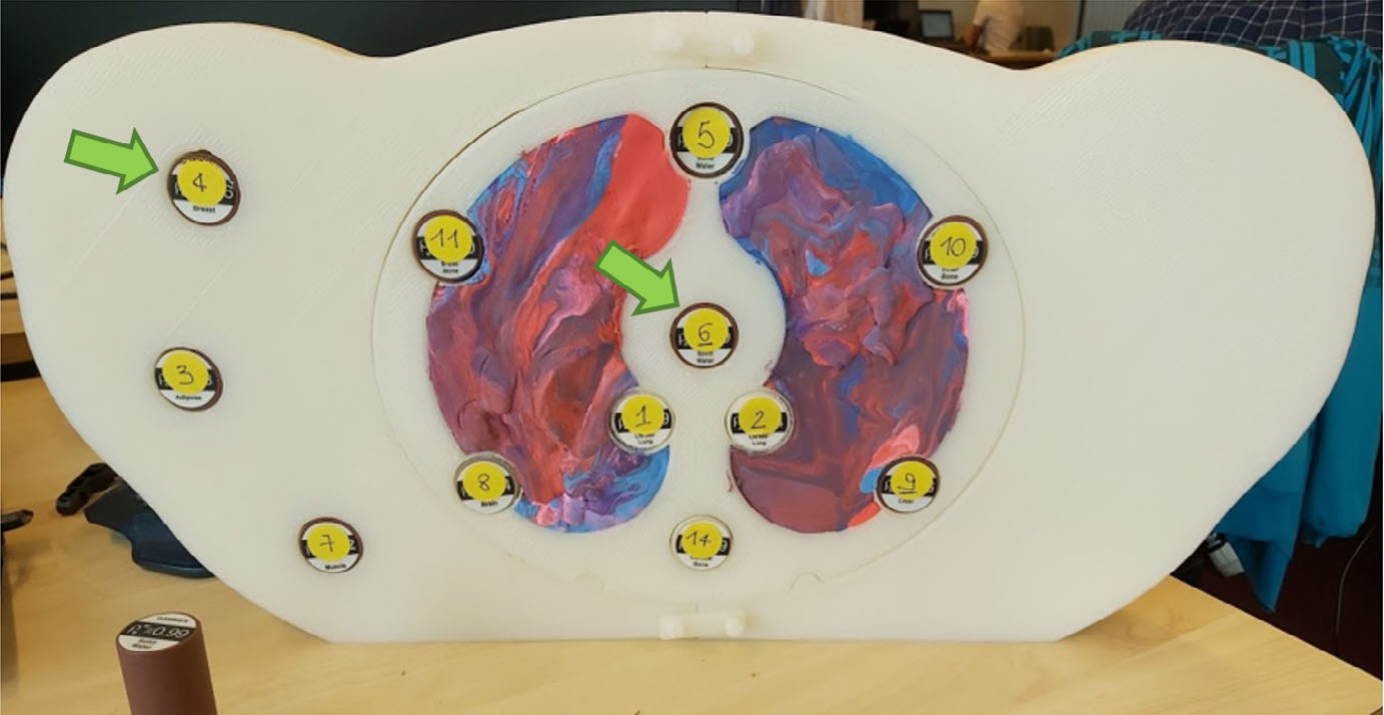
Three‐dimensional printed phantom (ground truth) for extended field‐of‐view evaluation. Green arrows show the position where tissue‐mimicking inserts were placed for the HU evaluation.

The dimensions of the commercial phantoms available in our clinic do not require eFoV since they fit well within the sFoV and shifting a small phantom might not be accurate since the displacement of the phantom in addition to an empty region at the center of the scanner can lead to HU variations. A large phantom based on the dimensions of a CT scan of a breast cancer patient who required eFoV acquisition was designed and 3D printed in‐house to verify eFoV accuracy in a relevant clinical setup. The phantom (Fig. 4) was printed using PLA (Polylactide—(C3H4O2)n, ρ ≈ 1.1 g/cm^3^) with 27 cm height, 9 cm length, and with 56 cm width which exceeds the sFoV of 50 cm. A fused deposition modeling (FDM) printer was used and settings adjusted (data not shown) until a homogeneity superior to 20 HU (1STD) within a 1 cm^2^ area (sFoV) was achieved. The phantom has holes allowing the insertion of tissue‐mimicking inserts. The region representing the lungs was filled with a low‐density rubber that mimics lung HU.

#### 2.2.1 Geometrical accuracy

Acquisitions were performed with the CT couch at three different heights to evaluate reconstruction accuracy for different volumes within the eFoV. The phantom was initially positioned with its center at the isocenter (first acquisition) of the CT scanner and then shifted up by approximately 9 cm (second acquisition) followed by an additional 3 cm shift (third acquisition). A reference image was obtained by combining two CT acquisitions. First, the phantom was shifted to the right side of the CT scanner so the left side of the phantom was within the sFoV. Second, the acquisition was repeated shifting the phantom to the left side of the CT scanner. The reconstructions of the right and left part of the phantoms were then combined to create a reference image of the phantom that consists only of measured data within sFoV. The reference image was rigidly registered to the images acquired at different couch heights and used to compare and analyze the performance of the two eFoV reconstruction algorithms HDFoV and HDeepFoV in terms of geometrical accuracy. Therefore, a binary mask was created from the reference image as well as from the HDFoV and HDeepFoV reconstructions. The binary masks equal 1 in regions that belong to the phantom area. All other areas in the binary mask were set to 0 to exclude the patient table and the air region from the evaluations. Based on the binary masks of the reference, the geometrical accuracy was evaluated quantitatively by computing the so‐called Jaccard Conformity Indices. The Jaccard Conformity Index is computed by dividing the intersecting area of two regions by the union of the regions. Accordingly, the Jaccard Conformity Index is 1 when there is a perfect match between two regions and it is 0 if two regions do not have any overlap. For the computation of the Jaccard Conformity Index the sFoV region was excluded and was computed only based on phantom areas that lie within the eFoV region.

A qualitative evaluation of the performance of HDFoV and HDeepFoV was done by visual inspection and comparison of the central phantom slices of the reference data set with the according slices in the HDFoV and HDeepFoV reconstructions. The central slice was taken here to avoid influences of the sharp transitions from air to phantom which may influence the image quality, especially for HDFoV since this algorithm uses data from adjacent slices to reconstruct the given slice (see Section 2.A.1).

#### 2.2.2 HU accuracy

HUs were evaluated using tissue‐mimicking inserts (GAMMEX, Middleton, USA) equivalent to breast, muscle, adipose tissue, solid water, cortical bone, inner bone, liver, inhale, and exhale lung. The inserts were placed at the top‐left position (Fig. 4) and the center of the phantom (used as reference HU value for each insert). Image acquisition was performed for each insert using approximately our current image acquisition protocol for breast cancer patients (120 kV, 250 mA (CareDose for dose reduction is used for patients, but was turned off for phantom measurements), 3 mm slice thickness, 0.35 pitch (clinical acquisitions use values around 1.2), Beam Hardening Correction, reconstruction kernel Qr40f (global filter, applied in projection domain), Admire iterative reconstruction algorithm strength 3, FoV of 780 mm). Images were reconstructed with HDFoV and HDeepFoV. Acquisitions were performed with the couch at two different heights so the top left inserts were once completely inside the eFoV and once completely inside the sFoV. The HU of bulk material (left side of the phantom) within the eFoV was compared against the reference image obtained within sFoV.

### 2.3 Clinical cases

Five radiotherapy patient cases for which eFoV reconstruction was necessary were selected to compare the reconstruction algorithms. The cases are representative of our clinical routine and were qualitatively evaluated by five radiotherapy physicians and five medical physicists. The clinical staff saw both reconstructions but were unaware of which eFoV algorithm was used to reconstruct the images and were asked to evaluate the images by indicating which algorithm provided more realistic results considering body boundaries. In addition, participants provided some remarks about image quality.

## 3 RESULTS

### 3.1 Geometrical accuracy

**Fig. 5:**
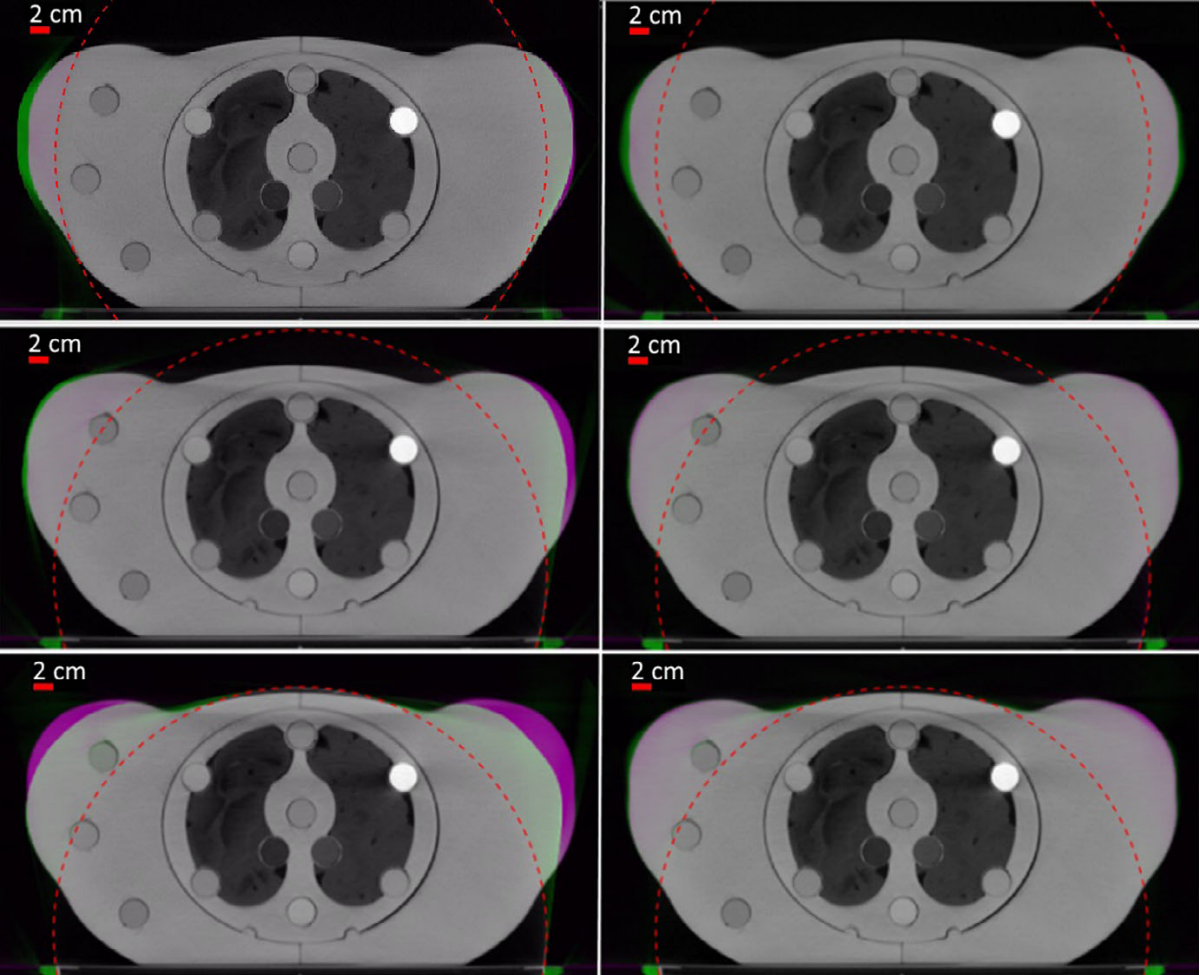
Computed tomography reconstructions for three different couch heights obtained with HDFoV (left column) and HDeepFoV (right column) compared against the reference image (top row is the lowest couch position). Purple and green regions indicate differences in HU values. Green regions around the boundaries indicate the phantom reconstructed with extended field‐of‐view algorithms is larger than the reference phantom while purple regions around the boundaries indicate the reference phantom is larger than represented with extended field‐of‐view reconstructions. All images were acquired with 3 mm slice thickness and reconstructed using the Qr40 kernel and a 78 cm FoV.

Figure 5 shows CT slices (approximately at the middle slice of the phantom) obtained using HDFoV and HDeepFoV for three different couch heights compared to the reference images. The shape of the phantom in the images varies with the volume of the phantom outside the sFoV for the images obtained with HDFoV while phantom boundaries (the region outside the dashed lines) are more consistent for HDeepFoV images. HDeepFoV showed the worst results for the lowest couch height (top row) with maximum differences below 1.0 cm. HDeepFoV accuracy increased for the remaining heights with maximum deviations below 0.5 cm while deviation for HDFoV are up to 2.5 cm (bottom row).

**Fig. 6:**
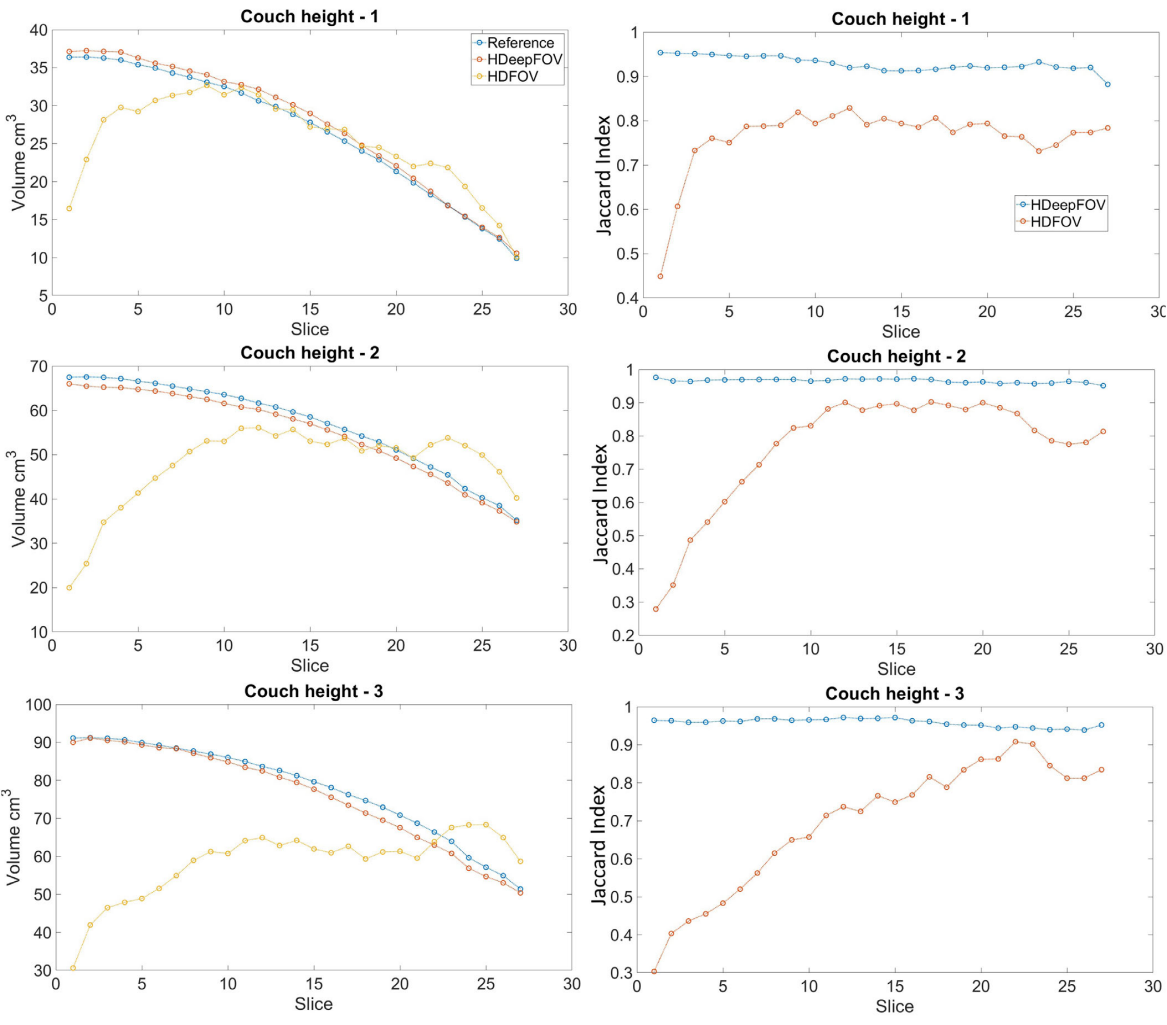
Volume with extended field‐of‐view per slice (left column) and Jaccard index per slice (right column) for three couch positions. Jaccard quantifies the similarities between two contours by dividing the intersection by the union of the volumetric contours with 1 representing a perfect match (100% overlap) and 0 indicating no overlap. The legend shown in the first row applies to all the figures in the same column. Lines connecting the points were added to guide the eyes.

Deviations from the reference geometry vary with the slice position and volume within eFoV as shown in Fig. 6. The slice volume within eFoV goes from ≈10 cm^3^ to ≈90 cm^3^ depending on the slice and couch position. The root‐mean‐square deviations (RMSDs) of the volume over all the slices and couch positions are 1.6 cm^3^ (HDeepFoV) and 18.8 cm^3^ (HDFoV). The mean Jaccard Conformity Indices over all the slices and couch positions are 0.95 ± 0.02 and 0.74 ± 0.15 for HDeepFoV and HDFoV, respectively.

### 3.2 HU accuracy

Table I shows the mean differences between HU values of the tissue‐mimicking inserts in the lateral positions within sFoV and within the eFoV (top position) compared against the reference values obtained with the inserts at the center of the phantom and isocenter of the CT scanner. HUs depend on the insert position within the sFoV with up to 145 HU variations for cortical bone (Lateral position within sFoV) compared to the obtained value at the isocenter. Absolute deviations for the cortical bone insert within eFoV reached 345 HU (HDFoV) and 277 HU (HDeepFoV). Deviation for lung inserts was also significant (>180 HU) for HDFoV while a better agreement (<60 HU) was observed for HDeepFoV. Variations are less significant (<80 HU) for soft tissue for both sFoV and eFoV.

**Table I:** HU difference between the computed tomography (CT) inserts at the lateral position within sFoV and extended field‐of‐view (eFoV) (table lifted) compared against the reference values obtained with the inserts at the center of the phantom and isocenter of the CT. The HU were averaged over five consecutive slices including voxels within a 1 cm radius (axial view) around the center of the insert.

	Lateral position within sFoV		Lateral position within eFoV	
	HDFoV	HDeepFoV	HDFoV	HDeepFoV
INHALED LUNG	−10.8	−4.3	228.6	−17.8
EXHALED LUNG	−9.9	−3.7	182.2	−59.1
ADIPOSE	−21.5	−13.7	78.5	18.9
BREAST	−24.4	−18.0	62.6	−1.4
S. WATER	−26.4	−20.0	49.0	−0.3
MUSCLE	−30.5	−22.5	34.3	−40.2
LIVER	−35.1	−28.0	19.1	−33.5
INNER BONE	−56.6	−48.7	−27.9	−72.4
CORTICAL BONE	−145.2	−137.6	−345.1	−277.1

**Fig. 7:**
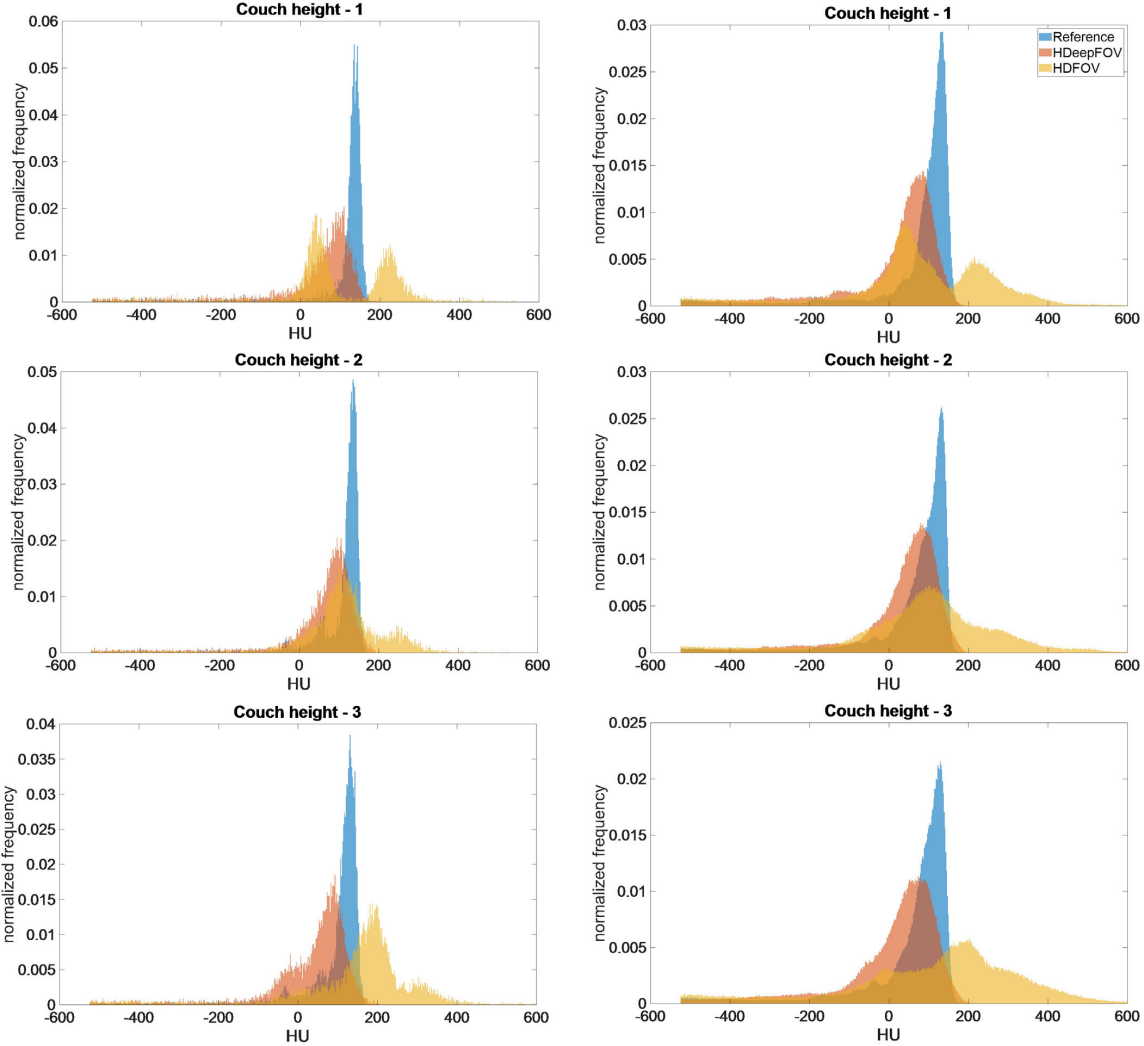
HU distributions for volume within extended field‐of‐view (eFoV) reconstructed with HDFoV and HDeepFoV compared to reference values obtained within sFoV (shifting the phantom). The left column shows the result for slice 13 (shown in Fig. 5) while results for the whole volume within eFoV are shown in the right column.

Figure 7 shows the HU distribution within the eFoV for the 3D printed material (side of the phantom without tissue‐mimicking inserts—see Fig. 4) compared against the reference values (obtained within the sFoV by shifting the phantom). The left column shows results obtained for the slice shown in Fig. 5 while HU distributions for the whole volume within the eFoV are shown in the right column. Both HDeepFoV and HDFoV have a wider HU distribution compared to the reference. HDeepFoV shows a systematic shift toward low HU values (≈60 HU shift in the position of the peak). HDFoV HU values go up to 600 HU while reference and HDeepFoV values are below 200 HU.

### 3.3 Clinical cases

**Fig. 8:**
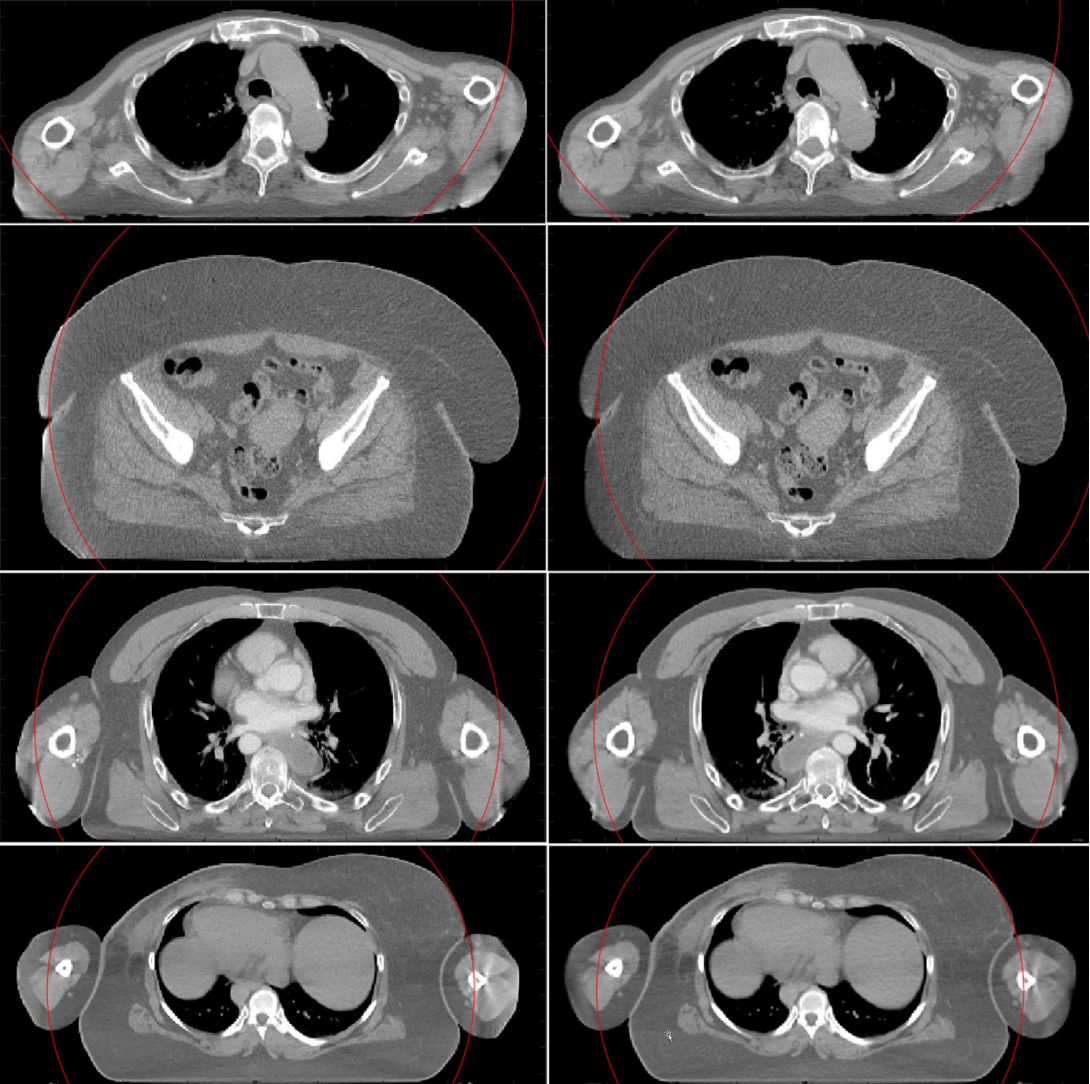
Clinical cases reconstructed using HDFoV (left) and HDeepFoV (right). The red circle shows sFoV boundaries.

The images obtained with HDeepFoV have superior quality compared to HDFoV for all patients as stated by 10 of 10 evaluators. Figure 8 shows some of the cases for which improvements were noticeable and image artifact reduction was explicitly mentioned by the evaluators. Overall, HDeepFoV images have fewer artifacts and better‐defined edges.

**Fig. 9:**
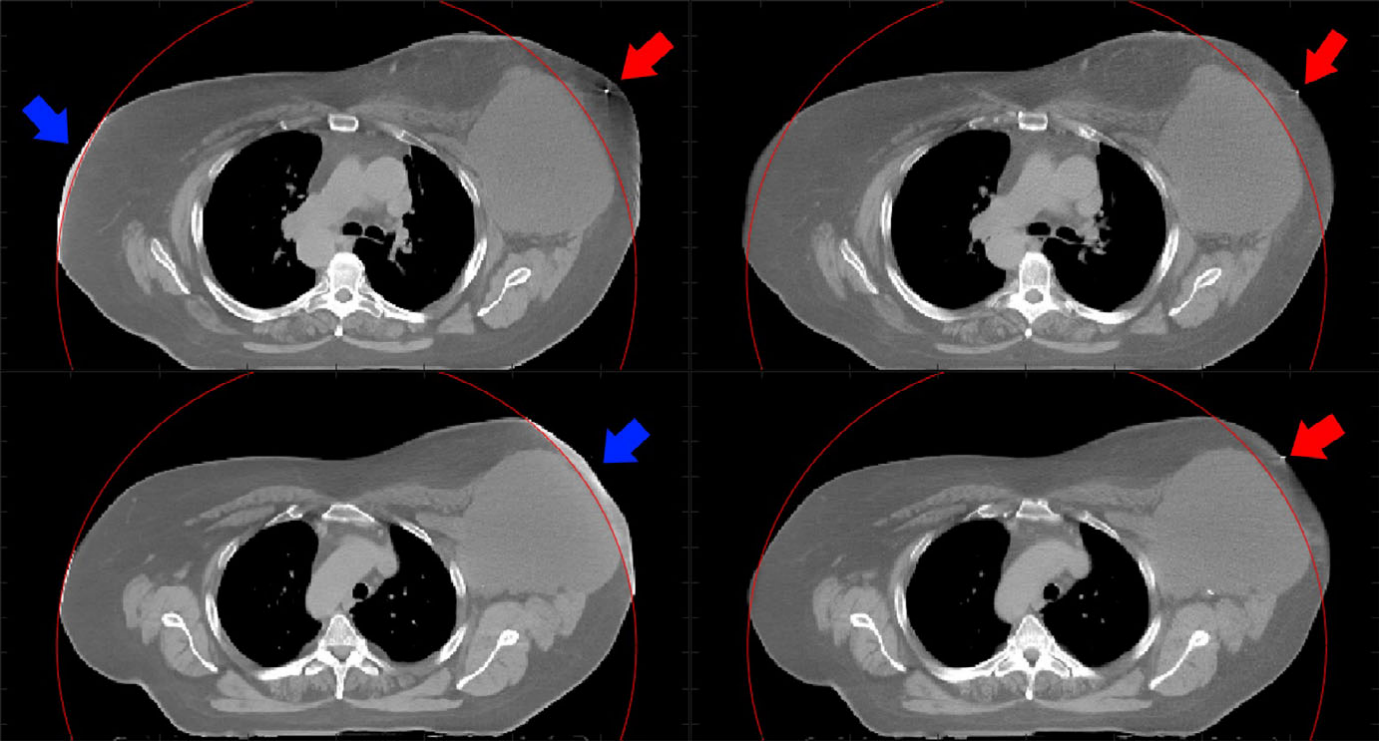
Reconstructed images using HDFoV (left column) and HDeepFoV (right column) for a breast cancer patient. The blue arrows indicate regions with high HU values near the limits of the sFoV. Red arrows point to a radiopaque marker placed on the skin. Note that the marker is shown within the body (top left) or not visible (bottom left) in the HDFoV reconstruction while the HDeepFoV (right column) shows the marker on the skin. The red circle shows sFoV boundaries.

Images reconstructed with HDFoV show a significant HU increase when the boundaries lie close to the sFoV limits as shown in Fig. 9 (blue arrows—left column). Differences greater than 100 HU were observed between the surrounding tissue and the volume within the eFoV. This issue was not observed in the HDeepFoV reconstructions (Fig. 9 –second column). A radiopaque marker placed at the skin of the patient is reconstructed inside the body in the HDFoV reconstruction (Fig. 9—red arrows) while HDeepFoV images show the marker more credibly placed on the surface of the patient.

## 4 DISCUSSION

Results obtained with the 3D printed phantom indicate the novel HDeepFoV algorithm has superior accuracy and robustness compared to HDFoV. Visual inspection of a central slice (Fig. 5) shows that the phantom boundaries (HDeepFoV) are similar regardless of the couch position while larger differences (up to 2.5 cm) were observed for the HDFoV reconstruction. Therefore, differences of up to a few centimeters (HDFoV) were reduced to a few millimeters (HDeepFoV).

A central slice of a custom phantom has been extensively evaluated by Cheung et al.,^12^ but no publications evaluated multiple slices with different volumes within eFoV. The gradual volume variation due to the smooth contour of the phantom used in this study shows that HDFoV performance varies considerably depending on the volume within eFoV and slice position. The reconstructed volume (HDFoV) for the first and second slice is less than half of the reference volume (Fig. 6). The eFoV volume obtained with HDFoV shows a similar agreement than volumes obtained using HDeepFoV only for a few central slices (Fig. 6—couch height 1). However, accurate volume prediction does not necessarily result in a large overlap with the reference volume. The Jaccard index of HDFoV reconstruction is always less than HDeepFoV even when the reconstructed volume within eFoV is similar for both algorithms (Fig. 6).

The Jaccard indices (Fig. 6) show a good agreement (0.95) with no apparent dependence on the slice location nor couch position for the HDeepFoV reconstruction. On the other hand, Jaccard indices for the HDFoV reconstruction show a strong dependence on slice location and couch position with indices varying from ≈0.3 to ≈0.9 with no clear pattern. A worse performance was expected for the HDFoV near the superior–inferior limits of the phantom since the reconstruction algorithm performs a tri‐dimensional interpolation so the limited length of the phantom impacts the performance of the algorithm. However, even the central slices show considerable differences in the reconstructed volume within eFoV due to the phantom geometry and couch height.

HU analysis also indicates a better agreement between HDeepFoV reconstructions and reference values with HDFoV reconstruction showing a much large HU spread. Although tissue mimicking inserts are commonly used for CT,^12^, ^27^ there are no reports of HU accuracy using these inserts in the eFoV region. Results indicate HU variation depending on the position within sFoV with small HU differences (≤35 HU) for low density materials and up to 145 HU deviation for the cortical bone insert. Differences are more significant within eFoV with HU variations for soft tissues below 79 HU and reaching 229 HU (HDFoV—lung) and −345 HU (HDFoV—cortical bone). HDeepFoV showed significant improvements for lung inserts with a maximum difference below 60 HU. The RMSD considering all the inserts within sFoV is 56 HU (HDFoV) and 51 HU (HDFoV) that can be considered equivalent. RMSD increases within eFoV reaching 156 HU (HDFoV) and 99 HU (HDeepFoV).

Results obtained with the 3D printed phantom are consistent with observations made for the patient cases. HU histograms (Fig. 7) show peaks below and above reference values for HDFoV that can also be observed for the patient images in Fig. 8 (left column—eFoV region) as low and high HU streaks within the eFoV. HDeepFoV reconstruction (Fig. 8—right column) has a more homogeneous HU distribution. In addition, unrealistic high HU values were observed for the phantom (Fig. 7) and a patient (Fig. 9—left column) when boundaries were very close to the eFoV limit. Therefore, additional caution is recommended when using HDFoV reconstruction with patient boundaries close to the sFoV limits.

Cheung et al.^12^ evaluated previous versions of eFoV reconstruction algorithms using a custom phantom with tissue‐mimicking inserts indicating the volume of the phantom within eFoV affects HU accuracy within the sFoV.^12^ Although eFoV reconstruction should not affect the measured data within the sFoV region, HU variations can be caused by the inaccuracies introduced by the detruncation algorithms which are then propagated into the sFoV region by the convolution step of filtered backprojection type of algorithms. It is not possible to provide a direct HU (eFoV) comparison against the results obtained by Cheung et al. since the author provided averaged values over the phantom material (eFoV) that differs from the material and geometry of the phantom used in this study. Nevertheless, image artifacts such as unrealistic high HU values near the edges of the sFoV (Fig. 9—HDFoV) are also visible. Cheung et al.^12^ reported high intensity artifacts around 150 HU which is consistent with the HDFoV results obtained in this manuscript. This issue has been reduced with the proposed HDeepFoV algorithm.

This study focused on HU and geometrical accuracy using a 3D printed phantom thicker than the CT scanner detector to account for the 3D interpolation required by the HDFoV reconstruction. The smooth contour of the phantom, mimicking a patient, allowed an evaluation of different volumes within eFoV. Although the obtained results provide relevant information about the accuracy and robustness of the methods, it is not possible to infer the dosimetric impact in radiotherapy plans (out of the scope of this work). It is expected that eFoV inaccuracies would be less relevant for highly modulated treatments^28^ with several fields from which only a few would cross the eFoV region than for treatments with a reduced number of treatment fields. Therefore, dose differences are patient and plan specific. A fictitious plan using the phantom as a reference cannot be directly extrapolated to patient cases for which there is no ground truth. 3D printing technology could support future studies aiming to address the dosimetric impact of the eFoV.

HDeepFoV reconstructions were considered superior by all experts that evaluated patient images. Although there are good indications (e.g., more uniform HU distribution, realistic contours, and the correct position of a CT marker on the skin (Fig. 9)), there is no ground truth for patient cases so the evaluation relies on the expertise of the medical staff being subject of interpretation.

## 5 CONCLUSION

There are applications and situations—especially in radiation therapy planning—where it simply is not possible to position the whole patient inside the sFoV of the CT scanner be it due to a special positioning of the patient or due to the size of the patient. To be able to reconstruct images outside the sFoV of the CT scanner there is no alternative but to use some kind of extrapolated data. To decide whether a given new algorithm for an extended field‐of‐view reconstruction is superior to the state‐of‐the‐art solution it is desirably that a ground truth (e.g., using realistic 3D printed phantoms) of the test images exists which then allows for a fair and reasonable comparison of the algorithms. To further increase the confidence in new algorithms—especially in the case of eFoV reconstruction where the data outside the sFoV of the scanner are an educated guess done by an algorithm—it is desirable that the algorithms can be tested in a close‐to‐clinical scenario with scanned data and not only with simulations. To achieve this we first developed a thorax phantom based on clinical data using the methods of 3D printing. We then used this phantom to investigate the performance of a novel deep learning‐based algorithm for eFoV reconstruction (HDeepFoV) quantitatively. HDeepFoV showed superior performance of the quantitative measurements and was also judged more accurate by medical experts based on patient data. Therefore, HDeepFoV may be a step further toward a more accurate planning base for radiation therapy.

## Funding information

This project was financially supported by Siemens Healthineers.

## Data Availability

The authors confirm that the data supporting the findings of this study are available within the article. Additional information (RAW data that support the findings of this study) can be made available on reasonable request from the corresponding author, G.P. Fonseca.
